# On the identity of *Chamaedrilus glandulosus* (Michaelsen, 1888) (Clitellata, Enchytraeidae), with the description of a new species

**DOI:** 10.3897/zookeys.501.9279

**Published:** 2015-04-30

**Authors:** Svante Martinsson, Emilia Rota, Christer Erséus

**Affiliations:** 1 Systematics and Biodiversity, Department of Biological and Environmental Sciences, University of Gothenburg, Box 463, SE-405 30 Göteborg, Sweden; 2 Department of Physics, Earth and Environmental Sciences, University of Siena, Via P.A. Mattioli 4, IT-53100 Siena, Italy

**Keywords:** *Cognettia*, *Chamaedrilus*, cryptic species, Oligochaeta, taxonomy

## Abstract

The taxonomy of *Chamaedrilus glandulosus* (Michaelsen, 1888) s. l., most commonly known previously as *Cognettia glandulosa*, is revised. A recent molecular systematic study has shown that this taxon harbours two cryptic, but genetically well separated lineages, each warranting species status. In this study these two lineages are scrutinized morphologically, on the basis of Michaelsen’s type material as well as newly collected specimens from Central and Northern Europe. *Chamaedrilus glandulosus* s. s. is redescribed and *Chamaedrilus varisetosus* **sp. n.** is recognized as new to science. The two species are morphologically very similar, differing mainly in size, but seem to prefer different habitats, with *Chamaedrilus glandulosus* being a larger aquatic species, and *Chamaedrilus varisetosus* being smaller and mainly found in moist to wet soil.

## Introduction

In 1888 Michaelsen described an enchytraeid worm, Pachydrilus sphagnetorum var. glandulosus Michaelsen, 1888, as a variant of *Pachydrilus sphagnetorum* Vejdovský, 1878. The description was based on material from the banks of the Bille and Elbe rivers in Hamburg, northern Germany. These two taxa were then transferred to *Marionina* Michaelsen, 1890 (in Pfeffer 1890), and Pachydrilus sphagnetorum var. glandulosus was considered a good species, *Marionina glandulosa*, separate from *Marionia sphagnetorum* (Michaelsen 1900). Later Friend (1919) assigned both species to *Chamaedrilus* Friend, 1913, an action seldom noticed by subsequent authors. For instance, when Nielsen and Christensen (1959) established *Cognettia*, they transferred *Marionina glandulosa* to their new genus without considering its previous placement in *Chamaedrilus*. Nielsen and Christensen’s (1959) concept of *Cognettia* came to embrace a number of terrestrial and freshwater enchytraeids and until recently it has been widely accepted. However, as noted by Schmelz and Collado (2010) and now more closely investigated by ourselves (Martinsson et al. 2014), *Cognettia* is indeed a junior synonym to *Chamaedrilus*. For details about the complex taxonomical history and a formal revision of *Chamaedrilus*, see Martinsson et al. (2014).

Several cryptic forms have been found within well-known morphology-based taxa of former *Cognettia* (Martinsson and Erséus 2014). The morphospecies *Chamaedrilus sphagnetorum* s. l. was found to be a non-monophyletic assemblage of at least four species; these have been revised and described by Martinsson et al. (2014). The taxon *Chamaedrilus glandulosus*, on the other hand, traditionally distinguished from *sphagnetorum* by the possession of secondary septal glands and longer spermathecal ectal ducts (Nielsen and Christensen 1959), was shown by both nuclear and mitochondrial DNA evidence to consist of two separately evolving lineages in Northern Europe. These two lineages appeared as sister species, i.e., representing a monophyletic group (Martinsson and Erséus 2014). According to Christensen (1959) *Chamaedrilus glandulosus* s. l. reproduces both by fragmentation and parthenogenetically, but the eggs must be activated by spermatozoa for normal development (Christensen 1961). However it is still possible that at least one of the two cryptic species occasionally reproduces biparentally. Uniparental reproduction makes species delimitation problematic, in particular when referring to the biological species concept (Mayr 1942). However, as discussed by Martinsson and Erséus (2014), asexual organisms form distinct clusters and can be delimited using the unified species concept by de Queiroz (2007). According to this concept, the sole requirement of a species is that it is a separately evolving metapopulation lineage, and criteria (e.g. morphological differences, reproductive isolation, or gene tree monophyly) from any of the more traditional species concepts can be used to delimit the lineages. The greater the number of criteria supporting a divergence, the stronger the case is for speciation, but, even a single piece of evidence, if properly substantiated, may be enough to establish lineage separation.

The aim of this study is to revise the taxonomy of *Chamaedrilus glandulosus* s. l. by delimiting *Chamaedrilus glandulosus* s. s., with the designation of a lectotype, and describing *Chamaedrilus varisetosus* sp. n.

## Material and methods

This study is based on two syntypes of Pachydrilus sphagnetorum var. glandulosus Michaelsen, 1888, from the original syntype series of ten, borrowed from the Zoological Museum of Hamburg University (ZMUH), Germany, of which one is here designated as lectotype, plus material analysed by Martinsson and Erséus (2014), and new specimens collected in northern and central Europe. A list of all examined specimens, with locality data and GenBank accession numbers for DNA-barcodes is given in Table 1.

**Table 1.** List of material included in this study, with specimen identification numbers, voucher numbers, collection data, GPS coordinates, and GenBank accession numbers for COI barcodes. Voucher numbers in bold indicate type specimens, barcode numbers in bold are newly generated sequences. Locality data are given in the form: country, province, municipality and locality; GPS coordinates are given as decimal degrees. CZ = Czech Republic, FIN = Finland, GER = Germany, NOR = Norway, SWE = Sweden.

Species	Spm. nos.	Museum voucher nos.	Sexual maturity	Collection locality	Coordinates		Leg.	Coll. date	Barcode Acc. nos.
					N	E			
*Chamaedrilus glandulosus*		**ZMUH V 429a**	mature	**GER. Hamburg**, Hamburg, Bille River bank	53.54	10.09	W. Michaelsen	Pre 1888	-
*Chamaedrilus glandulosus*		**ZMUH V 429b**	immature	**GER. Hamburg**, Hamburg, Bille River bank	53.54	10.09	W. Michaelsen	Pre 1888	-
*Chamaedrilus glandulosus*	CE2011	SMNH133613	immature	**SWE. Västergötland**, Vårgårda, Lången Lake littoral	57.9973	12.5868	C. Erséus	Jun 30 2006	KF672372
*Chamaedrilus glandulosus*	CE2841	SMNH133614	immature	**SWE. Öland**, Borgholm, Räpplinge, stream	56.8195	16.9444	A. Ansebo, L. Matamoros & C. Erséus	Jun 13 2007	KF672374
*Chamaedrilus glandulosus*	CE2887	SMNH133615	submature	**SWE. Södermanland**, Vingåker, Låttern Lake littoral	59.0854	16.0426	C. Erséus	Jul 30 2007	KF672375
*Chamaedrilus glandulosus*	CE2888	SMNH133616	submature	**SWE. Södermanland**, Vingåker, Låttern Lake littoral	59.0854	16.0426	C. Erséus	Jul 30 2007	KF672376
*Chamaedrilus glandulosus*	CE2889	SMNH133617	immature	**SWE. Södermanland**, Vingåker, Låttern Lake littoral	59.0854	16.0426	C. Erséus	Jul 30 2007	KF672377
*Chamaedrilus glandulosus*	CE2890	SMNH133618	immature	**SWE. Södermanland**, Vingåker, Låttern Lake littoral	59.0854	16.0426	C. Erséus	Jul 30 2007	KF672378
*Chamaedrilus glandulosus*	CE2891	SMNH133619	immature	**SWE. Södermanland**, Vingåker, Låttern Lake littoral	59.0854	16.0426	C. Erséus	Jul 30 2007	KF672379
*Chamaedrilus glandulosus*	CE8510	SMNH133620	immature	**SWE. Lappland**, Kiruna, Abisko, marsh pond	68.3485	18.9719	D. Fontaneto	Jul 6 2007	JN260143
*Chamaedrilus glandulosus*	CE10655	SMNH133612	immature	**FIN. Keski-Suomi**, Jyväskylä, Alvajärvi Lake littoral	62.315	25.730	H. Saarikoski	Fall 2009	JN260270
*Chamaedrilus glandulosus*	CE17761	SMNH142041	immature	**SWE. Södermanland**, Vingåker, Hjälmaren Lake littoral	59.133	15.814	C. Erséus	Jul 27 2012	**KP878475**
*Chamaedrilus glandulosus*	CE17806	SMNH142042	immature	**SWE. Södermanland**, Vingåker, Hjälmaren Lake littoral	59.133	15.814	C. Erséus	Jul 27 2012	**KP878476**
*Chamaedrilus glandulosus*	CE18516	SMNH142043	submature	**SWE. Västergötland**, Lerum, Aspen Lake littoral	57.7656	12.2525	C. Erséus & B. Williams	Jun 1 2013	-
*Chamaedrilus glandulosus*	CE18517	SMNH142044	mature	**SWE. Västergötland**, Lerum, Aspen Lake littoral	57.7656	12.2525	C. Erséus & B. Williams	Jun 1 2013	**KP878477**
*Chamaedrilus glandulosus*	CE18518	SMNH142045	immature	**SWE. Västergötland**, Lerum, Aspen Lake littoral	57.7656	12.2525	C. Erséus & B. Williams	Jun 1 2013	**KP878478**
*Chamaedrilus glandulosus*	CE20212	SMNH142046	immature	**NOR. Östfold**, Halden, Enningdalselva River	58.9099	11.5210	C. Erséus	Oct 12 2013	**KP878474**
*Chamaedrilus varisetosus*	CE2634	SMNH133600	immature	**SWE. Öland**, Borgholm, S Greda, sandy soil	56.9929	16.8765	A. Ansebo, L. Matamoros & C. Erséus	Jun 12 2007	KF672367
*Chamaedrilus varisetosus*	CE2931	SMNH133601	immature	**SWE. Öland**, Borgholm, Egby, peaty soil	56.8621	16.8539	A. Ansebo, L. Matamoros & C. Erséus	Jun 12 2007	KF672368
*Chamaedrilus varisetosus*	CE4027	SMNH133602	immature	**SWE. Skåne**, Ystad, Nyvangsskogen, wet soil	55.5606	13.8239	C. Erséus	May 31 2008	KF672369
*Chamaedrilus varisetosus*	CE4028	SMNH133603	immature	**SWE. Skåne**, Ystad, Nyvangsskogen, wet soil	55.5606	13.8239	C. Erséus	May 31 2008	KF672370
*Chamaedrilus varisetosus*	CE6626	SMNH133604	immature	**SWE. Uppland**, Vallentuna, Brottby,peaty soil	59.5477	18.2467	C. Erséus	Jun 4 2009	KF672371
*Chamaedrilus varisetosus*	CE9376	SMNH133605	immature	**SWE. Medelpad**, Timra, Söråker, forest soil	62.5235	17.4782	C. Erséus	Jun 8 2010	KF672424
*Chamaedrilus varisetosus*	CE9517	SMNH133606	immature	**SWE. Lappland**, Kiruna, Björkliden, peat	68.4262	18.3509	C. Erséus	Jun 12 2010	JN260194
*Chamaedrilus varisetosus*	CE9524	SMNH133607	immature	**SWE. Lappland**, Kiruna, Björkliden, river	68.4277	18.4448	C. Erséus	Jun 12 2010	KF672425
*Chamaedrilus varisetosus*	CE9525	SMNH133608	immature	**SWE. Lappland**, Kiruna, Björkliden, river	68.4277	18.4448	C. Erséus	Jun 12 2010	JN260195
*Chamaedrilus varisetosus*	CE9526	SMNH133609	immature	**SWE. Lappland**, Kiruna, Björkliden, river	68.4277	18.4448	C. Erséus	Jun 12 2010	JN260282
*Chamaedrilus varisetosus*	CE9536	SMNH133610	immature	**SWE. Lappland**, Kiruna, Kiruna, forest soil	67.8546	20.2173	C. Erséus	Jun 13 2010	JN260198
*Chamaedrilus varisetosus*	CE9581	SMNH133611	immature	**SWE. Lappland**, Vilhelmina, Klimpfjäll, grassland soil	65.0621	14.8066	C. Erséus	Jun 15 2010	JN260206
*Chamaedrilus varisetosus*	CE11485	SMNH142026	immature	**SWE. Västergötland**, Lerum, Almekärr, wet soil	57.7614	12.2706	C. Erséus & A. Achurra	Apr 23 2011	**KP878462**
*Chamaedrilus varisetosus*	CE18904	SMNH142027	immature	**NOR. Telemark**, Hjartdal, Kovstulheia, stream	59.8182	8.7222	C. Erséus & B. Williams	Jun 13 2013	**KP878463**
*Chamaedrilus varisetosus*	CE19031	SMNH142028	immature	**NOR. Telemark**, Kviteseid, Kviteseid, wet forest litter	59.3532	8.5196	C. Erséus & B. Williams	Jun 13 2013	**KP878460**
*Chamaedrilus varisetosus*	CE19052	**ZMBN99905**	mature	**NOR. Buskerud**, Hol, Örtedalsåna River, wet moss	60.4866	7.8562	C. Erséus	Aug 10 2013	**KP878464**
*Chamaedrilus varisetosus*	CE19113	SMNH142029	immature	**NOR. Buskerud**, Hol, Geilo, forest soil	60.5329	8.2113	C. Erséus	Aug 11 2013	**KP878465**
*Chamaedrilus varisetosus*	CE19117	SMNH142030	immature	**NOR. Buskerud**, Hol, Geilo, forest soil	60.5329	8.2113	C. Erséus	Aug 11 2013	**KP878466**
*Chamaedrilus varisetosus*	CE19677	SMNH142031	immature	**NOR. Sör-Tröndelag**, Tydal, Langsvola, litter	62.8388	11.805	C. Erséus	Aug 14 2013	**KP878467**
*Chamaedrilus varisetosus*	CE19716	SMNH142032	immature	**NOR. Sör-Tröndelag**, Röros, Hitterdalen, stream bank	62.6060	11.6599	C. Erséus	Aug 15 2013	**KP878461**
*Chamaedrilus varisetosus*	CE19749	SMNH142033	immature	**NOR. Sör-Tröndelag**, Röros, Doktortjönna Lake shore	62.5763	11.3745	C. Erséus	Aug 15 2013	**KP878468**
*Chamaedrilus varisetosus*	CE19818	**ZMBN99906**	submature	**NOR. Hedmark**, Engerdal, Nymoen, wet moss	61.6569	11.8164	C. Erséus	Aug 15 2013	**KP878469**
*Chamaedrilus varisetosus*	CE19819	**SMNH Type-8732**	submature	**NOR. Hedmark**, Engerdal, Nymoen, wet moss	61.6569	11.8164	C. Erséus	Aug 15 2013	**KP878470**
*Chamaedrilus varisetosus*	CE19823	SMNH142034	immature	**NOR. Hedmark**, Engerdal, Nymoen, wet moss	61.6569	11.8164	C. Erséus	Aug 15 2013	**KP878471**
*Chamaedrilus varisetosus*	CE19831	SMNH142035	immature	**NOR. Hedmark**, Engerdal, Nymoen, wet moss	61.6569	11.8164	C. Erséus	Aug 15 2013	**KP878472**
*Chamaedrilus varisetosus*	CE19832	SMNH142036	immature	**NOR. Hedmark**, Engerdal, Nymoen, wet moss	61.6569	11.8164	C. Erséus	Aug 15 2013	**KP878459**
*Chamaedrilus varisetosus*	CE20021	SMNH142037	immature	**NOR, Östfold**, Hvaler, Asmalöy, dry soil	59.0630	10.9396	C. Erséus	Sep 22 2013	**KP878473**
*Chamaedrilus varisetosus*	CE20046	SMNH142038	immature	**NOR. Östfold**, Fredikstad, Trosvik, litter on clay	59.2364	10.9012	C. Erséus	Sep 23 2013	**KP878479**
*Chamaedrilus varisetosus*	SM171	SMNH142039	immature	**CZ. NW Moravia**, Okres Šumperk, Králický Sněžník, moss in stream	50.1499	16.8624	K. Elliott & S. Martinsson	Jun 15 2013	**KP878457**
*Chamaedrilus varisetosus*	SM172	SMNH142040	immature	**CZ. NW Moravia**, Okres Šumperk, Králický Sněžník, moss in stream	50.1499	16.8624	K. Elliott & S. Martinsson	Jun 15 2013	**KP878458**

Newly collected specimens were DNA-barcoded using the cytochrome c oxidase subunit I (COI) marker, as described by Martinsson and Erséus (2014); DNA was extracted from a few posterior-most segments of each worm, using Epicentre QuickExtract DNA Extraction Solution 1.0, following the manufacturer’s instructions, while the rest of the specimen was used for morphological studies, i.e., as a voucher. All new barcodes were matched with COI sequences of *Cognettia glandulosa* ‘A’ and ‘B’ from Martinsson and Erséus (2014). For tissue samples of the over 100 years old syntypes, newly designed primers were tested to amplify a short part of COI, as well as a fragment of the ribosomal 16S mtRNA gene, respectively, but these attempts were unsuccessful.

**Figure 1.**
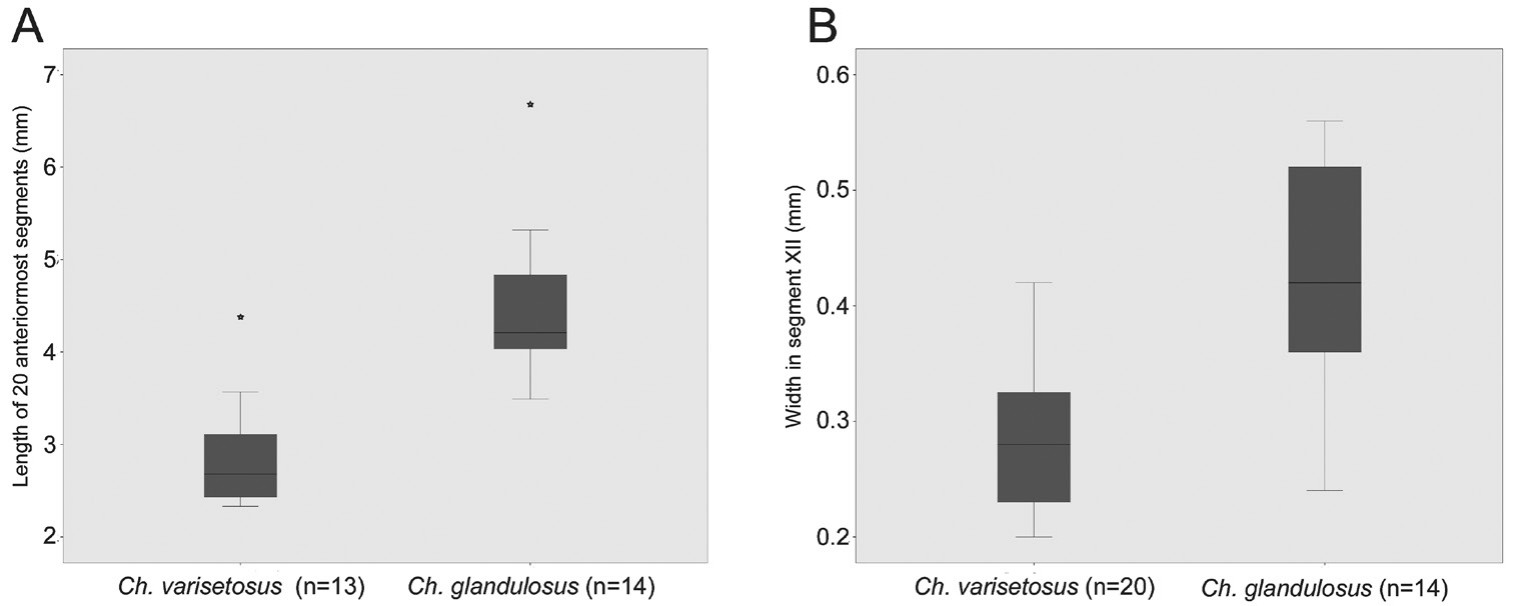
Boxplots showing differences in body size between *Chamaedrilus glandulosus* (Michaelsen, 1888) sensu stricto and *Chamaedrilus varisetosus* sp. n. **A** Length of 20 anteriormost segments **B** Width in segment XII. Both differences are significant (two-sided t-tests; P = 1.5E-5 and P = 5.5E-5, respectively).

Unless otherwise mentioned in the descriptions, all information refers to the studied material only, in that the two taxa treated in this paper have previously been classified as one and the same species. Michaelsen’s syntypes were first studied as temporary mounts in glycerol. The newly designated lectotype was then stained with paracarmine and permanently mounted in Canada balsam on a slide as outlined by Erséus (1994), and so were all other voucher specimens (including the types of *Chamaedrilus varisetosus* sp. n.). All measurements and observations were made on preserved and somewhat compressed animals under a compound microscope (Leitz Laborlux K). As the posterior parts of the specimens were used for DNA extraction, the body size is arbitrarily given as the length of the 20 anteriormost segments and the width in segment XII (latter representing not clitellum but general body width). This size estimate was used also in Martinsson et al. (2014). In the descriptions, body measurements are given as the range followed by the mean ± 1 standard deviation. Differences in size between the two species were visualised with boxplots (Fig. 1, where asterisks denote the outliers), and tested by using two-sided t-tests performed in SPSS v. 22 (SPSS Inc., Chicago). Sketches were drawn using a camera lucida and used as templates for producing digital illustrations with Adobe PhotoShop.

The geographical distributions consider the origin of our material as well as that of COI barcode matches in BOLD (Barcoding of Life Data Systems, Ratnasingham and Hebert 2007). The Barcode Index Numbers (BIN) (Ratnasingham and Hebert 2013) are given under Remarks, for respective species. The BIN system clusters the sequences to produce operational taxonomic units that are assumed to closely correspond to species (http://www.boldsystems.org).

All specimens studied, including new types, are deposited in the Swedish Museum of Natural History (SMNH), Stockholm, the University Museum Bergen (UMB), Norway, and the Zoological Museum Hamburg (ZMUH), Germany; all COI barcodes are deposited in GenBank (see Table 1).

## Taxonomy

### Chamaedrilus glandulosus

Taxon classificationAnimaliaEnchytraeidaEnchytraeidae

(Michaelsen, 1888) sensu stricto

Fig. 2

**Figure 2.**
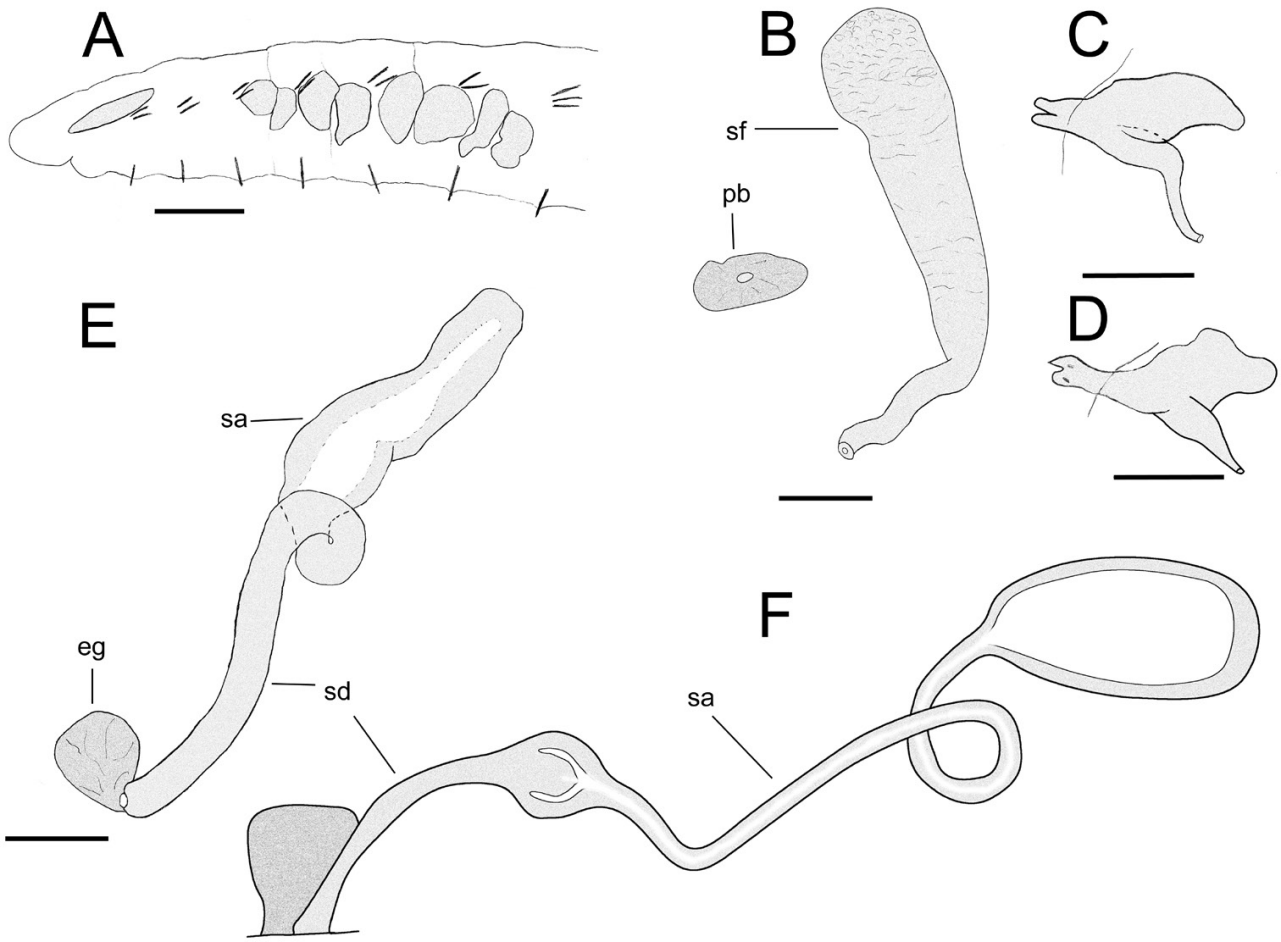
*Chamaedrilus glandulosus* (Michaelsen, 1888) sensu stricto. **A** Anterior part of body (immature specimen) in lateral view, indicating chaetal distribution and the size, shape and number of pharyngeal glands **B** Sperm funnel, ental tract of vas deferens and penial bulb, to show their relative size proportions **C** Nephridium at septum 8/9, lateral view **D** Nephridium at septum 10/11, lateral view **E** Spermatheca **F** Spermatheca redrawn from Michaelsen (1888). Abbreviations: eg = ectal gland; pb = penial bulb; sa = spermathecal ampulla; sd = spermathecal duct; sf = sperm funnel. Scale bars: 200 µm (**A**); 50 µm (**B–E**).

Pachydrilus sphagnetorum var. glandulosus Michaelsen, 1888: 490, plate 23, fig. 2a–c.
Marionia sphagnetorum var. glandulosa ; Michaelsen 1889: 29.
Marionina glandulosa ; Michaelsen 1900: 74.
Chamaedrilus glandulosus ; Friend 1919: 174, partim.
Enchytraeoides glandulosa ; von Bülow 1955: 257.
Cognettia glandulosa ; Nielsen and Christensen 1959: 43, fig. 30, partim; Schmelz and Collado 2010: 79, partim.
Cognettia glandulosa B; Martinsson and Erséus 2014.

#### Lectotype.

ZMUH V 429a, mature anterior part, in alcohol, leg. W. Michaelsen, date not given (before 1888).

#### Type locality.

**GERMANY: Hamburg**, banks of Bille River, in detritus (“*Billeufer, im Detritus*”) (N 53.54°, E 10.09°).

#### Paralectotype.

ZMUH V 429b, immature specimen, in alcohol; same collection data as for lectotype.

#### Additional type material

**(not studied). Paralectotypes** ZMUH V 429b, 8 specimens in alcohol, same collection data as for lectotype.

#### Other material.

See Table 1. In total 15 specimens, of which 1 from Finland, one from Norway and 13 from Sweden (whereof one mature and three submature). All specimens except one are DNA barcoded (Table 1).

#### Diagnosis.

Can be separated from all other European species of *Chamaedrilus* except *Chamaedrilus varisetosus* by its unique combination of 2–4 pairs of well-developed secondary pharyngeal glands, two chaetae per lateral bundle in preclitellar segments, and three chaetae in all other bundles, spermathecae with comparatively long ectal ducts, and genitalia shifted forward 3–4 segments (in relation to normal placement in Enchytraeidae). No characters completely separate this species from *Chamaedrilus varisetosus* sp. n., but specimens of *Chamaedrilus glandulosus* are usually larger and have only two chaetae in the lateral bundles of preclitellar segments, whereas *Chamaedrilus varisetosus* usually has three chaetae in lateral bundles of III-V. Furthermore, *Chamaedrilus glandulosus* is found in aquatic habitats only (i.e. submerged under water for most of the time), whereas *Chamaedrilus varisetosus* is found in both aquatic and terrestrial habitats; so far we have not found them occurring together.

#### Description.

EXTERNAL CHARACTERS: Size: length of 20 anteriormost segments 3.49-6.68 mm, mean 4.55±0.87 (n=11); body width in XII 0.24–0.56 mm, mean 0.42±0.10 (n = 14). Chaetae sigmoid without nodulus, 60–100 µm long, chaetal formula 2,(3)-3:3-3, with 3 lateral chaetae per bundle from VII-IX; in sexually mature specimens, ventral chaetae, or both ventral and lateral chaetae, missing in the segment bearing male pores (VIII or IX). In the sexually mature and submature specimens examined, clitellum poorly developed.

INTERNAL CHARACTERS: Brain concave posteriorly, 160–210 µm long. Pharyngeal glands 3–4 primary pairs; 2–4 pairs of well-developed secondary glands (Fig. 2A), secondary glands behind the first pair of primary glands sometimes missing. Dorsal blood vessel arising in XVI–XX. First pair of nephridia present at 7/8–8/9; nephridia with efferent duct originating antero-ventrally, close to septum; anteseptale consisting of funnel only; postseptale elongate (Fig. 2C–D). Chloragogen cells granulated; 35–55 µm long. Coelomocytes granulated, round to oval, 25–30 µm long.

Seminal vesicle distinct and unpaired in one specimen (CE18516), poorly developed in all other mature or submature specimens. Other genitalia paired. Sperm funnel about 200 µm long, tapering, 25 µm wide basally, 50 µm wide proximally; collar 55–60 µm wide. Spermatozoa on collar in a few mature/submature worms. Vas deferens long, simple, with several loops, about 12 µm wide. Penial bulb poorly developed, about 25 µm wide, 60–65 µm long (Fig. 2B). Male pores in VIII or IX. Spermathecae paired; pores located slightly below lateral chaetae; ectal duct smooth, 240 µm long, about 17 µm wide; ectal gland 35–40 µm in diameter; ampulla oval, about 150 µm long, not attached to oesophagus (Fig. 2E); sperm in ampulla of lectotype only. Spermathecae confined to V or entering into VI.

#### Habitat and distribution.

Occurs in freshwater habitats, in sand and gravel bottoms in lakes and small streams, and climbing on vegetation and dead wood in water. Barcoded specimens document occurrence in Finland, Germany, Norway and Sweden, but the species is probably more widely distributed, not only in Europe. For instance, *Chamaedrilus glandulosus* s. l. has also been reported from North America: the records by Nurminen (1973) and Healy (1996) are insufficiently described and cannot even tentatively be assigned to any of the two species, and the records by Schlaghamerský (2013) and Schlaghamerský et al. (2014) are likely to be *Chamaedrilus varisetosus*, see under Habitat and distribution for that species.

#### Biology.

Seems to reproduce mainly parthenogenetically; specimens with developing genitalia are found from June to July (Sweden).

#### Remarks.

Michaelsen (1888; 1900) described this species as sturdier than *Chamaedrilus sphagnetorum*, with 2 chaetae per preclitellar lateral bundle and three chaetae in all other bundles. This together with the fact that Michaelsen’s type material was collected at an aquatic site makes us confident that our new material is conspecific with Michaelsen’s species. Michaelsen (1888) described the spermathecae *in vivo* as very long (“they often project, in spite of much meandering, up to the segment VII”) and the ampullae to consist each of an ectal enlargement followed by a long connecting tube and an expanded ental chamber (Fig. 2F). In our new material the spermathecae seem to be either not fully developed or much contracted after fixation: they show simple oval ampullae, not differentiated into ectal and ental compartments. In the mature lectotype we can only follow the spermathecae to what we interpret as the ampullar ectal enlargement. *Chamaedrilus glandulosus* is larger than *Chamaedrilus varisetosus* described below. Both the length of the 20 anteriormost segments (P = 1.5E-5) and the width in segment XII (P = 5.5E-5) differ significantly between the two species (Fig. 1).

This species is represented in BOLD by BIN: AAT8923.

### Chamaedrilus varisetosus sp. n.

Taxon classificationAnimaliaEnchytraeidaEnchytraeidae

http://zoobank.org/BEA27C2F-484B-465A-AA06-034E84F0FF20

Fig. 3

Chamaedrilus glandulosus ; Friend 1919: 174, partim.
Cognettia glandulosa ; Nielsen and Christensen 1959: 43, fig. 30, partim; Schmelz and Collado 2010: 79, partim.
Cognettia glandulosa A; Martinsson and Erséus 2014.

#### Holotype.

ZMBN99905, CE19052, mature, anterior part, COI barcode acc. no. KP878464, leg. Christer Erséus, Aug 10, 2013.

#### Type locality.

**NORWAY: Buskerud**, Hol, at Örtedalsåna River (S of Haugastöl), elevation 1,075 m above sea level (N60.4866°, E7.8562°).

#### Paratypes.

ZMBN99906, CE19818, submature, anterior part, COI barcode acc. no. KP878469; **NORWAY: Hedmark**, Engerdal, Nymoen at Femundelva (Trysilelva) River, at Nordre Husfloen Farm (N61.6569°, E11.8164°), leg. Christer Erséus, Aug 15, 2013. SMNH type-8723, CE19819, submature, anterior part, COI barcode acc. no. KP878470. Same collection data as for the other paratype.

#### Other material.

See Table 1. Twenty-seven immature specimens, of which 2 from the Czech Republic, 12 from Norway, and 13 from Sweden, all DNA-barcoded.

#### Etymology.

The species is named after the variation in numbers of chaetae in the lateral preclitellar bundles.

#### Diagnosis.

The new species can be separated from all other European species of *Chamaedrilus* except *Chamaedrilus glandulosus* s. s. by its unique combination of 3–4 pairs of well-developed secondary pharyngeal glands, two chaetae in most lateral bundles in preclitellar segments, and three chaetae in all other bundles, spermathecae with comparatively long ectal ducts, and genitalia shifted forward 3–4 segments (in relation to normal placement in Enchytraeidae). No characters completely separate this species from *Chamaedrilus glandulosus*, but specimens of *Chamaedrilus varisetosus* are generally smaller, have shorter chaetae and smaller internal organs, and usually have a few preclitellar lateral bundles with three chaetae (*Chamaedrilus glandulosus* constantly has two chaetae per lateral bundle in preclitellar segments). Furthermore, *Chamaedrilus varisetosus* is mainly found in moist to wet soils, whereas *Chamaedrilus glandulosus* is only found in aquatic habitats.

#### Description.

EXTERNAL CHARACTERS: Size: length of 20 anteriormost segments 2.33–4.38 mm, mean 2.89±0.59 (n = 13); body width in XII 0.20–0.42 mm, mean 0.28±0.07 (n = 20). Chaetae sigmoid without nodulus, 50–60 µm long, chaetal formula 2,3-(2),3:3–3; most specimens with 3 chaetae in lateral bundles of III(or IV)-V and 2 chaetae in the other lateral preclitellar bundles, but some specimens have 2 chaetae in all preclitellar lateral bundles; in sexually mature specimens, chaetae missing in the segment bearing male pores (VIII or IX). In the mature and submature specimens examined, clitellum only developed (but poorly) in the segment bearing the male pores and ½ a segment posterior and anterior to that segment.

**Figure 3.**
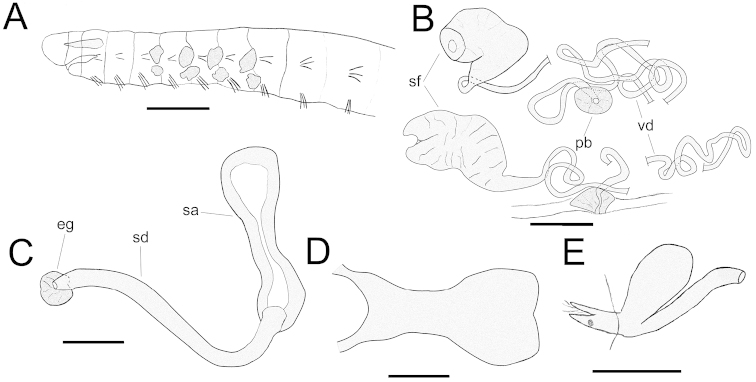
*Chamaedrilus varisetosus* sp. n. **A** Anterior part of body (immature specimen) in lateral view, indicating chaetal distribution and the size, shape and number of pharyngeal glands **B** Male genitalia of a mature worm with male pores in segment VIII **C** Spermatheca **D** Brain, dorsal view **E** Nephridium at septum 10/11, lateral view. Abbreviations: eg = ectal gland; pb = penial bulb; sa = spermathecal ampulla; sd = spermathecal duct; sf = sperm funnel; vd = vas deferens. Scale bars: 200 µm (**A**); 50 µm (**B-E**).

INTERNAL CHARACTERS: Brain slightly concave posteriorly, concave anteriorly, 125–140 µm long, about twice as long as broad (Fig. 3D). Pharyngeal glands, 3–4 primary pairs; 3–4 pairs of well-developed secondary glands (Fig. 3A), secondary glands behind the last pair of primary glands sometimes missing. Dorsal blood vessel arising in XIII–XVII, rarely in XI or XVIII. First pair of nephridia present at 8/9–11/12; nephridia with efferent duct originating antero-ventrally, close to septum; anteseptale consisting of funnel only; postseptale oval, elongate (Fig. 3E). Chloragogen cells granulated, 20–30 µm long. Coelomocytes finely granulated, round to oval, approximately 20 µm long.

Seminal vesicle unpaired, distinct in all three mature/submature specimens. Other genitalia paired. Sperm funnel about 100 µm long, 40–50 µm wide; collar indistinct, 25–30 µm wide. Spermatozoa not observed on collar. Vas deferens long, with several loops, about 5–7 µm wide. Penial bulb poorly developed, about 25 µm wide, 35–40 µm long (Fig. 3B). Male pores in VIII or IX. Spermathecae paired; pores located slightly below lateral chaetae; ectal duct smooth, 225 µm long, approximately 15 µm wide; ectal gland 25–30 µm in diameter; ampulla about 150 µm long, with ectal enlargement, followed by a contraction and a tubular to oval ental chamber; no sperm observed in ampulla; ampulla not attached to oesophagus (Fig. 3C). Spermathecae entering into VI.

#### Habitat and distribution.

Found both in aquatic and terrestrial habitats. In freshwater found on stony bottoms in rivers, on land found in both deciduous and coniferous forest as well as in grassland soils. Known from Canada (BOLD record), the Czech Republic, Finland (BOLD record), Norway and Sweden, but may be more widely distributed in Europe and North America. Schlaghamerský’s (2013) description of *Cognettia glandulosa* from Michigan fits our description of *Chamaedrilus varisetosus*. This and Schlaghamerský’s et al. (2014) records from Minnesota and Wisconsin are likely to refer to the same species.

#### Biology.

Parthenogenetic reproduction more limited in time (maturing specimens found in August in Norway) than fragmentation (observed in May-September in Sweden and Norway). Worms with regenerating tails and/or heads rather frequent. This species may correspond to the population studied by Christensen (1959), in which the number of mature worms was high for a short period during the autumn. The variation in number of the lateral chaetae corresponds to that given in the diagnosis by Nielsen and Christensen (1959).

#### Remarks.

This species is represented in BOLD by BIN: AAT9501.

## Discussion

The two species treated in this paper, *Chamaedrilus glandulosus* sensu stricto and *Chamaedrilus varisetosus* sp. n., are easily separated morphologically from other species of *Chamaedrilus* by a unique combination of characters: the secondary pharyngeal glands are well developed in several segments, there are two chaetae in most preclitellar lateral bundles, but no enlarged chaetae, the genital organs are shifted forwards, and the spermathecae have comparatively long ectal ducts. The two species are morphologically similar and they have therefore been regarded as a single taxon by previous authors (e.g., Nielsen and Christensen 1959; Schmelz and Collado 2010). As demonstrated in the present paper, they can only be separated by their body size, chaetal size (and prevailing number) and, when fully grown, by the proportions of most internal organs. Genetically, however, they are well separated from each other (Martinsson and Erséus 2014), and they are also ecologically separated, with *Chamaedrilus glandulosus* found in aquatic habitats, whereas *Chamaedrilus varisetosus* is predominantly found in moist to wet soil. Ecological and physiological differences have been found between cryptic lineages in morphospecies of various organisms (e.g. Beauchamp et al. 2002; Feckler et al. 2014; Sattler et al. 2007), and if such lineages are not formally recognized and named, the differences may continue to be overlooked or neglected.

Martinsson and Erséus (2014) found *Chamaedrilus glandulosus* and *Chamaedrilus varisetosus* sp. n. to be sister species, nested within a part of the *sphagnetorum*-complex, making the latter non-monophyletic. The *sphagnetorum*-complex also turned out to be morphologically more heterogeneous than *Chamaedrilus glandulosus* s. l. (Martinsson et al. 2014), which could probably be, at least partly, explained by its non-monophyly. However, not even the two morphologically indistinguishable species, *Chamaedrilus sphagnetorum* s. s. and *Chamaedrilus pseudosphagnetorum* Martinsson et al., 2014 came out as sister species in the phylogenetic study (Martinsson and Erséus 2014).

Without the genetic data, the delimitation of *Chamaedrilus glandulosus* and *Chamaedrilus varisetosus* would have been much more challenging, all the more so because these worms, like those in the *sphagnetorum* complex, are mostly found sexually immature. It should also be considered that these species, even when mature, actually reproduce uniparentally, as mentioned in the introduction and discussed earlier by Martinsson and Erséus (2014). Uniparental reproduction makes species delimitation harder; however, we still believe this is possible using the unifying species concept (see Introduction). In the present case, we have a combination of genetic, ecological and morphological differences, supporting the split of *Chamaedrilus glandulosus* s. l. into two species. It should further be noted that it is not known with certainty if Christensen (1959; 1961) studied both species, or only one of them. As mentioned in the description, *Chamaedrilus varisetosus* seems to correspond well with the taxon studied in his 1959 paper and also fits the description given by Nielsen and Christensen (1959). Until the mode(s) of reproduction is (are) studied again for the two species, we cannot exclude the possibility that one or both species may reproduce biparentally, at least occasionally.

Genetic studies discovering cryptic and unnoticed diversity need to be followed by formal taxonomic revision, including careful morphological scrutiny, updated descriptions and species names, if possible based on barcoded types. We believe that an integrative approach, combining genetic and morphological data with as much as possible of ecological and physiological information, will strengthen studies of enchytraeid systematics.

## Supplementary Material

XML Treatment for Chamaedrilus glandulosus

XML Treatment for Chamaedrilus varisetosus
