# Corrigenda: Martinsson S, Rota E, Erséus C (2015) On the identity of *Chamaedrilus glandulosus* (Michaelsen, 1888) (Clitellata, Enchytraeidae), with the description of a new species. ZooKeys 501: 1–14. doi: 10.3897/zookeys.501.9279

**DOI:** 10.3897/zookeys.504.9972

**Published:** 2015-05-19

**Authors:** Svante Martinsson, Emilia Rota, Christer Erséus

**Affiliations:** 1 Systematics and Biodiversity, Department of Biological and Environmental Sciences, University of Gothenburg, Box 463, SE-405 30 Göteborg, Sweden; 2 Department of Physics, Earth and Environmental Sciences, University of Siena, Via P.A. Mattioli 4, IT-53100 Siena, Italy

It has come to our attention that in the work referenced above Figures 3A and 3E are incorrect. The published figures showed two chaetae in all lateral preclitellar bundles of *Chamaedrilus varisetosus* sp. n., instead of three chaetae in segments III-V (which is the most common occurrence), and an incomplete nephridial efferent duct.

The correct, whole Figure 3 is reproduced here below.

**Figure 3.**
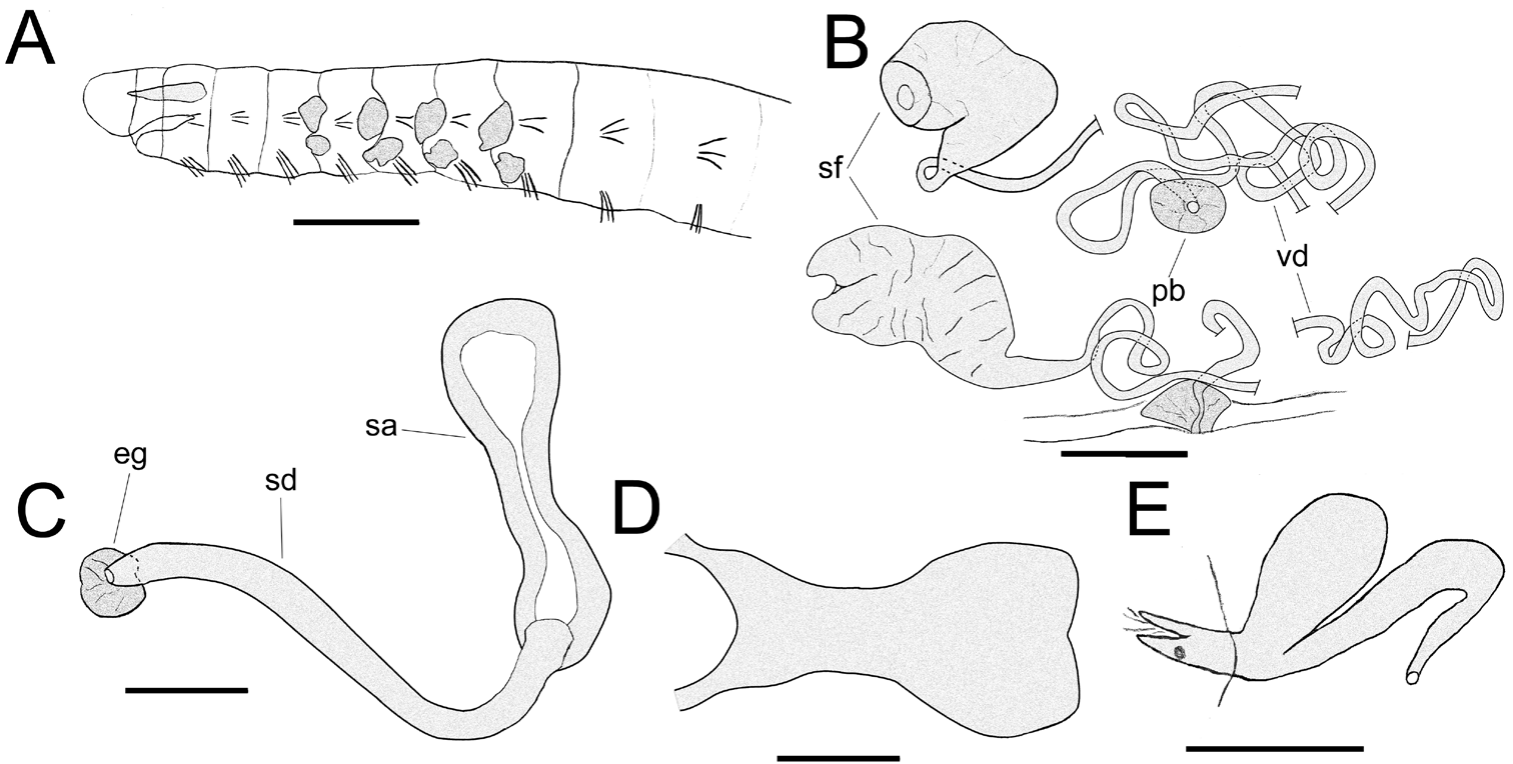
*Chamaedrilus varisetosus* sp. n. **A** Anterior part of body (immature specimen) in lateral view, indicating chaetal distribution and the size, shape and number of pharyngeal glands **B** Male genitalia of a mature worm with male pores in segment VIII **C** Spermatheca **D** Brain, dorsal view **E** Nephridium at septum 10/11, lateral view. Abbreviations: eg = ectal gland; pb = penial bulb; sa = spermathecal ampulla; sd = spermathecal duct; sf = sperm funnel; vd = vas deferens. Scale bars: 200 μm (**A**); 50 μm (**B–E**).

Furthermore, at the end of Material and methods, the University Museum Bergen should have been abbreviated ZMBN instead of UMB. We would like to thank Mark J. Wetzel (IHNS, University of Illinois) for bringing the incorrect abbreviation to our attention.

